# Undermining the cry for help: the phytopathogenic fungus *Verticillium dahliae* secretes an antimicrobial effector protein to undermine host recruitment of antagonistic *Pseudomonas* bacteria

**DOI:** 10.1111/nph.70686

**Published:** 2025-10-29

**Authors:** Anton Kraege, Wilko Punt, Andrea Doddi, Jinyi Zhu, Natalie Schmitz, Nick C. Snelders, Bart P. H. J. Thomma

**Affiliations:** ^1^ Institute for Plant Sciences, Cluster of Excellence on Plant Sciences (CEPLAS) University of Cologne 50674 Cologne Germany; ^2^ Department of Environmental Biology Sapienza University of Rome 00185 Rome Italy; ^3^ Theoretical Biology and Bioinformatics Group, Department of Biology University of Utrecht 3584CH Utrecht the Netherlands

**Keywords:** AMP, antimicrobial, intermicrobial interaction, microbe–microbe interaction, microbiota, plant pathogen

## Abstract

- During pathogen attack, plants recruit beneficial microbes in a ‘cry for help’ to mitigate disease development. Simultaneously, pathogens secrete effectors to promote host colonisation through various mechanisms, including targeted host microbiota manipulation.
- Inspired by *in silico* antimicrobial activity prediction, we investigated the antimicrobial activity of Av2, an effector of *Verticillium dahliae*, *in vitro*. Furthermore, its role in *V. dahliae* virulence was assessed through microbiota sequencing of inoculated plants, microbial co‐cultivation assays, and inoculations in a gnotobiotic plant cultivation system.
- Av2 appears structurally unique and lacks domains that hint towards its function. We show that Av2 inhibits bacterial growth and acts as a virulence factor during host colonisation. Microbiota sequencing revealed involvement of Av2 in suppression of *Pseudomonas* spp. recruitment upon plant inoculation with *V. dahliae*, indicating that Av2 suppresses the cry for help. We show that several *Pseudomonas* spp. are antagonistic to *V. dahliae* and sensitive to Av2 treatment.
- We conclude that *V. dahliae* secretes Av2 to suppress the plant's cry for help by inhibiting the recruitment of antagonistic *Pseudomonas* spp. to pave the way for successful plant invasion.

## Introduction

Plants associate with a plethora of microbes above and below ground, collectively called the microbiota, that can positively impact plant productivity and health (Berendsen *et al*., 2018; Trivedi *et al*., 2020). Through the secretion of root exudates, plants shape their microbiota and actively recruit beneficial microbes to mitigate biotic and abiotic stresses (López *et al*., 2008; Berendsen *et al*., 2018). Under pathogen attack, plants can modify these exudates to selectively attract protective microbes in order to limit disease progression. This targeted recruitment in response to pathogen infection is known as the plant's ‘cry for help’ (Berendsen *et al*., 2018; Yuan *et al*., 2018; Liu *et al*., 2024; Spooren *et al*., 2024). For example, cucumber plants increase the exudation of tryptophan during *Fusarium oxysporum* infection, which promotes the recruitment of beneficial *Bacillus amyloliquefaciens* that can mitigate disease progression (Liu *et al*., 2017).

Ultimately, the cry for help, which results in the recruitment of beneficial microbes, may have a legacy effect in cases when it leads to an increased population of these microbes in the soil, resulting in the establishment of disease‐suppressive soils that protect future plants grown in the same soil (Rolfe *et al*., 2019; Mesny *et al*., 2024). However, the development of such a legacy effect typically requires years and many plant generations to fully establish (Rolfe *et al*., 2019). Arguably, the most famous example of such a legacy effect concerns the decline of take‐all disease, caused by the fungal plant pathogen *Gaeumannomyces tritici*, in wheat over years of monoculture that has been associated with the recruitment of 2,4‐diacetylphloroglucinol‐producing *Pseudomonas* spp. (Raaijmakers & Weller, 1998).

To detect pathogens, plants have evolved a complex immune system that recognises a multitude of microbe‐derived molecules to activate appropriate defence responses (Jones & Dangl, 2006). Initial immune responses are triggered upon recognition of conserved microbe‐associated molecular patterns (MAMPs), such as chitin or flagellin, by plant membrane‐localised MAMP recognition receptors that activate pattern‐triggered immunity (PTI) responses (Jones & Dangl, 2006; Cook *et al*., 2015). In response, host‐adapted pathogens have evolved strategies to suppress or overcome such PTI responses, which include the secretion of virulence factors, also known as effectors (Rovenich *et al*., 2014). In turn, particular host genotypes evolved to recognise effectors, or their activities, by resistance (R) proteins that include cell surface and cytoplasmic receptors that activate effector‐triggered immunity (Jones & Dangl, 2006; Cook *et al*., 2015).

Most effectors characterised to date deregulate host immune responses or target other aspects of host physiology through various biochemical activities and mechanisms (Rovenich *et al*., 2014). For example, the effector Ecp6 is secreted by *Cladosporium fulvum* to sequester chitin oligosaccharides that are released from its cell walls to prevent recognition by chitin immune receptors (Sánchez‐Vallet *et al*., 2013). Intriguingly, several research groups have recently uncovered a novel function of effectors besides the modulation of host physiology by showing that several pathogens secrete effectors that target host‐associated microbiota through the display of selective antimicrobial activity in order to promote host colonisation (Snelders *et al*., 2020; Gómez‐Pérez *et al*., 2023).

Several antimicrobial effectors have been functionally characterised in the soil‐borne fungus *Verticillium dahliae*, a presumed asexual filamentous fungus that causes vascular wilt disease on hundreds of host plants (Fradin & Thomma, 2006). The fungus generates genetic diversity through large‐scale chromosomal rearrangements and segmental duplications, leading to hypervariable regions between *V. dahliae* strains that are called adaptive genomic regions (AGRs) (de Jonge *et al*., 2013; Faino *et al*., 2016; Cook *et al*., 2020). These AGRs are enriched in repeats and in effector genes and display a unique chromatin profile that sets these regions apart from core genomic regions (Cook *et al*., 2020). Interestingly, despite being dispersed across the genome, these AGRs were found to physically interact in the nucleus, possibly contributing to their differential behaviour (Torres *et al*., 2024). Overall, similar to other filamentous pathogens, *V. dahliae* has a compartmentalised genome‐containing AGRs with increased plasticity compared with core genomic regions, an observation often referred to as a ‘two‐speed genome’ (Raffaele & Kamoun, 2012; Torres *et al*., 2021).

The first *V. dahliae* effector for which antimicrobial activity was shown is the AGR‐encoded lineage‐specific effector Ave1 that was identified by comparative genomics between *V. dahliae* strains that are controlled by *Ve1‐*mediated resistance in tomato and resistance‐breaking strains that are virulent towards *Ve1* plants (de Jonge *et al*., 2012). Besides being recognised by the tomato Ve1 immune receptor as an avirulence factor, Ave1 contributes to fungal virulence on plants lacking *Ve1* by targeting antagonistic bacteria of the Sphingomonadales order (Snelders *et al*., 2020). Notably, Ave1 is not the only *V. dahliae* effector protein with antibacterial activity, as a search for effectors with homology to known antimicrobial proteins within the *V. dahliae* secretome yielded the AMP2 effector that is expressed in soil extract. AMP2 revealed complementary activity to Ave1, suggesting that *V. dahliae* exploits different effectors to cope with the diversity of microbial competitors in soil (Snelders *et al*., 2020). The antimicrobial activity of *V. dahliae* effector proteins is not restricted to bacteria, as the defensin‐like effector AMP3 was found to target the mycobiota (Snelders *et al*., 2021). Intriguingly, and in contrast to Ave1 and AMP2, AMP3 is exclusively expressed at late infection stages when resting structures are formed in decaying plant tissue while host immune responses fade and opportunists and fungal decay organisms invade host tissues (Snelders *et al*., 2021).

Over the years, only two *R* loci were identified that confer resistance against *V. dahliae* in tomato. Besides the recognition of Ave1 by the Ve1 receptor, the fungal effector Av2 is recognised in *V2* tomato plants, although the corresponding *R* gene has not yet been cloned (Usami *et al*., 2017; Chavarro‐Carrero *et al*., 2021). Similar to Ave1, Av2 is a small (73 amino acid mature protein; net charge +1.8) secreted protein produced only by a subset of *V. dahliae* strains. Apart from homologues found in other *Verticillium* spp., the only homologues of this effector were found in the *Fusarium* genus (Chavarro‐Carrero *et al*., 2021). *Verticillium dahliae Av2* occurs in two allelic variants that differ in one non‐synonymous single nucleotide polymorphism (SNP) that are both recognised in *V2* plants, and so far its intrinsic function for the pathogen has remained enigmatic (Chavarro‐Carrero *et al*., 2021). In this study, we aimed to characterise the virulence function of *Av2* through a combination of *in silico* and functional analysis.

## Materials and Methods

### Detection of *V. dahliae Av2* expression in soil extract

For each treatment, 10^6^ conidiospores of *Verticillium dahliae* Kleb. strain JR2 were added to 10 ml potato dextrose broth (PDB) and incubated while shaking with 130 rpm at 28°C for 2 d (Ecotron, Infors‐HT, Bottmingen, Switzerland). Subsequently, the mycelium was collected using sterilised miracloth (Merck, Darmstadt, Germany) and washed with sterilised water. Next, the mycelium was transferred to new flasks containing 10 ml of soil extract that was prepared by adding 40 g of potting soil (Balster Einheitserde, Frödenberg, Germany) to 200 ml of sterilised water followed by incubation at room temperature for 2 d, after which soil particles were pelleted by centrifugation for 30 min at 4000 ***g*** and the supernatant was collected. The flasks were then incubated while shaking with 130 rpm at 28°C for 5 d (Ecotron, Infors‐HT, Bottmingen, Switzerland). Next, mycelium was recollected using sterilised miracloth and washed with sterilised water. RNA was extracted using TRIzol reagent (Thermo Fisher Scientific, Waltham, MA, USA) of which 1 μg was transcribed into cDNA using the PrimeScript™ RT reagent Kit with gDNA Eraser (Takara Bio USA, San Jose, CA, USA). Real‐time PCR was performed using SsoAdvance Universal SYBR Green Supermix (BioRad, Hercules, CA, USA) on a CFX Opus Real‐Time PCR System (BioRad) and the expression of effector genes was normalised using the *V. dahliae* glyceraldehyde 3‐phosphate dehydrogenase gene (*VdGAPDH*) as a reference.

### Heterologous expression of Av2 homologues

The *Av2* alleles encoding *V. dahliae* Av2 and Av2^V73E^ (from strains TO22 and JR2, respectively) and their homologues from *Fusarium oxysporum* f. sp. *pisi* and *F. redolens* (*Fop*Av2 and *Fr*Av2, respectively) were codon‐optimised for expression in *E. coli* and cloned into the pET15b vector (Merck, Darmstadt, Germany) such that the proteins are produced without a signal peptide and as a fusion protein with an N‐terminal His_6_ tag. All vectors were ordered from BioCat GmbH (Heidelberg, Germany). While *Vd*Av2 and Av2^V73E^ were produced in *E. coli* strain BL21 (Thermo Fisher Scientific), *Fop*Av2 and *Fr*Av2 were produced in *E. coli* strain SHUFFLE T7 (New England Biolabs, Ipswich, MA, USA). A preinoculum of bacterial cultures was incubated overnight in Lysogeny broth (LB) supplemented with 50 μg ml^−1^ ampicillin at 37°C for BL21 and at 30°C for SHUFFLE T7 while shaking at 170 rpm (Ecotron, Infors‐HT, Bottmingen, Switzerland). Subsequently, the preinoculum was transferred to 1 l of LB supplemented with ampicillin (50 μg ml^−1^) and incubated at 37°C (BL21) or 30°C (SHUFFLE T7) until the OD_600_ reached 0.6–0.8. Next, isopropyl‐1‐thio‐β‐d‐galactopyranoside (IPTG) was added to a final concentration of 1 mM, and the culture was incubated for 4 h at 37°C (BL21) or 30°C (SHUFFLE T7). Next, cells were pelleted through centrifugation (21 000 ***g***) at 4°C and resuspended in 6 M guanidine, 10 mM TRIS–HCl, and 10 mM β‐mercaptoethanol (pH 8.0) and incubated overnight at 4°C while rotating continuously. After centrifugation at 21 000 ***g*** for 30 min, proteins were purified from the supernatant by immobilised metal affinity chromatography (IMAC) on a custom‐packed 5 ml Ni^2+^ CYTIVIA column (XK16/20 Column, Cytiva, Marlborough, MA, USA) with His60 Ni Superflow Resin (Takara Bio USA, San Jose, CA, USA). Fractions containing the protein of interest were identified by sodium dodecyl sulphate‐polyacrylamide gel electrophoresis (SDS‐PAGE) analysis, combined, and dialysed in a stepwise fashion. To this end, the protein was dialysed in a stepwise manner over several 18‐h intervals. Initially, dialysis was performed against 4 M guanidine, 50 mM BIS‐TRIS, 10 mM reduced glutathione, and 2 mM oxidised glutathione (pH 7.0). This was followed by dialysis against 3 M guanidine, 50 mM BIS‐TRIS, 10 mM reduced glutathione, and 2 mM oxidised glutathione (pH 6.5). Subsequently, the protein was dialysed against 2 M guanidine, 100 mM BIS‐TRIS, 250 mM ammonium sulfate, 10 mM reduced glutathione, and 2 mM oxidised glutathione (pH 6.5), followed by 1 M guanidine, 100 mM BIS‐TRIS, 125 mM ammonium sulfate, 10 mM reduced glutathione, and 2 mM oxidised glutathione (pH 5.8). The final dialysis step was performed in 100 mM BIS‐TRIS, 125 mM ammonium sulfate, 10 mM reduced glutathione, and 2 mM oxidised glutathione (pH 5.8). Ultimately, the protein was dialysed against potassium phosphate buffer (pH 6.5). Final protein concentrations were determined with Nanodrop (Thermo Fisher Scientific) based on absorbance at 280 nm and adjusted to a final concentration of 16 μM.

### *In vitro* antimicrobial activity assays

All bacteria used in the assays originated from a tomato culture collection (Punt *et al*., 2025). After growth of bacterial isolates on tryptone soy agar (TSA) at 28°C, single colonies were selected and grown overnight in tryptone soy low salt broth (TSB LS) at 28°C while shaking at 200 rpm (Ecotron, Infors‐HT, Bottmingen, Switzerland). Subsequently, optical density was measured at 600 nm (OD_600_) and adjusted to 0.05 by dilution with TSB LS. One hundred microlitres of bacterial culture were mixed with 100 μl of Av2 protein variants (16 μM) in clear 96‐well plates (BRAND SCIENTIFIC GMBH, Wertheim, Germany) with three replicates for each treatment. The plates were incubated in a CLARIOstar® plate reader (BMG LABTECH, Ortenberg, Germany) at 28°C with double orbital shaking every 15 min (10 s at 300 rpm), after which the OD_600_ was measured (Snelders *et al*., 2020).

### Plant disease assays

Inoculation of tomato plants to determine the virulence of *V. dahliae* was performed as described previously (Fradin *et al*., 2009). Briefly, conidiospores of *V. dahliae* wild‐type or *Av2* deletion strain (Chavarro‐Carrero *et al*., 2021) were harvested after 10 d of cultivation on potato dextrose agar (PDA). The conidiospore suspensions were centrifuged at 10 000 ***g*** for 10 min, and the pellets were resuspended in water. This washing step was repeated twice before spores were counted, and the concentration was adjusted to 10^6^ conidiospores ml^−1^. For the inoculation, 10‐d‐old tomato (*Solanum lycopersicum* L.) MoneyMaker plants were carefully uprooted, roots were rinsed in water, and placed into the inoculum for 6 min. Next, plants were placed back into soil and placed in the glasshouse at 22°C during 16 h : 8 h day/night periods with a maximum of 80% relative humidity, and symptoms were monitored at 14 d post inoculation (dpi). To this end, canopy areas were measured and fungal biomass inside the tomato stem was determined. For the latter, samples were frozen in liquid nitrogen, ground to a fine powder, and DNA was isolated using phenol–chloroform extraction (Chavarro‐Carrero *et al*., 2021). *Verticillium dahliae* biomass was quantified through real‐time PCR using *V. dahliae*‐specific primers targeting the internal transcribed spacer (ITS) region of the ribosomal DNA (Snelders *et al*., 2020). The tomato *Rubisco* gene was used for sample calibration (Snelders *et al*., 2020).

### Tomato stem microbiota sequencing

Tomato stem samples were collected, washed with sterile water, frozen in liquid nitrogen, and manually ground using a mortar and pestle. Total DNA was extracted following a phenol–chloroform‐based extraction procedure (Chavarro‐Carrero *et al*., 2021), and DNA concentrations were determined using a Qubit fluorometer (Thermo Fisher Scientific). Sequence libraries were prepared following amplification of the V5–V7 region of the bacterial 16S rDNA (799F and 1139R) as described previously (Wippel *et al*., 2021) and sequenced (paired‐end 300 bp) on an Illumina MiSeq V3 Platform (Cologne Center for Genomics, Cologne, Germany). Sample barcoding was done as described previously (Fadrosh *et al*., 2014).

### Microbiota analysis

Sequencing data were processed using R v.4.2.0 as described previously (Callahan *et al*., 2016; Snelders *et al*., 2020). Briefly, reads were demultiplexed using cutadapt (v.4.1; Martin, 2011). Afterwards reads were trimmed and filtered to an average paired read length of 412 bp with the Phred score of 30. From the trimmed reads, OTUs were inferred using the DADA2 method (v.1.24; Callahan *et al*., 2016). Taxonomy was assigned using the Ribosomal Database Project (RDP, v.18; Cole *et al*., 2014). The pyloseq package (v.1.40.0; McMurdie & Holmes, 2013) was used to calculate β‐diversity (weighted unifrac distance) after the data was normalised with the metagenomeseq package (v.1.38.0; Paulson *et al*., 2013) using cumulative sum scaling. PERMANOVA was performed with the vegan (v.2.6‐4; Oksanen *et al*., 2004) package. Differential abundance analysis was done using the DESeq2 package (v.1.36.0; Love *et al*., 2014) using a negative binomial Wald test and a significance *P*‐adjusted threshold < 0.05.

### Homology analysis of Av2

In order to find functional domains within the Av2 sequence, the online version of InterProScan was used with the amino acid sequence of Av2. Structural prediction of Av2 was done using AlphaFold2 (v.2.0 Jumper *et al*., 2021) with default settings. To find structural homologues, the Av2 structure was queried against the AlphaFold structural database (Varadi *et al*., 2022) using the Foldseek search server (van Kempen *et al*., 2023). Protein surface charge was calculated and visualised using PyMOL (The PyMOL Molecular Graphics System, v.3.0 Schrödinger, LLC) using the APBS electrostatics plugin (Jurrus *et al*., 2018).

### *In vitro* competition assay

Conidiospores of *V. dahliae* strain TO22 and the *VdAv2* deletion strain were harvested from a PDA plate using sterile water and diluted to a concentration of 2 × 10^6^ conidiospores ml^−1^ in ½‐strength Murashige & Skoog (½MS) medium (Duchefa, Haarlem, the Netherlands). Plant‐associated *Pseudomonas* spp. (Punt *et al*., 2025) were cultured overnight in ½MS medium. Next, overnight cultures were adjusted to OD_600_ of 0.05 in ½MS and added to the conidiospores, and 500 μl of the microbial mixture was added into a 12‐well flat bottom polystyrene tissue culture plate (Sarstedt, Nümbrecht, Germany). Following 48 h of incubation at RT with shaking at 150 rpm (Ecotron, Infors‐HT, Bottmingen, Switzerland), genomic DNA was extracted using the SmartExtract DNA kit (Eurogentec, Maastricht, the Netherlands), and *V. dahliae* biomass was quantified through real‐time PCR using *V. dahliae*‐specific primers targeting the ITS region of the ribosomal DNA (Snelders *et al*., 2020). A spike‐in DNA sequence was added during DNA extraction for sample calibration (Guo *et al*., 2020). Genomic sequences of the tested *Pseudomonas* spp. (Punt *et al*., 2025) were used to infer rooted species trees based on single‐copy orthologous genes (Emms & Kelly, 2019).

### Gnotobiotic tomato cultivation for *V. dahliae* inoculations

For tomato cultivation, a previously developed Flowpot system was used (Kremer *et al*., 2021; Punt *et al*., 2025). A 1:1 blend of potting soil (Balster Einheitserdewerk, Fröndenberg, Germany) and vermiculite (LIMERA Gartenbauservice, Geldern‐Walbeck, Germany) was autoclaved three times on a liquid cycle and filled into 50 ml syringes (Terumo Europe, Leuven, Belgium). To check for substrate sterility, 500 mg of substrate was added to 10 ml of 100 mM MgCl_2_ and shaken at 300 rpm at room temperature for 1 h (Ecotron, Infors‐HT, Bottmingen, Switzerland). Subsequently, the samples were diluted 1000‐fold, and 50 μl of the dilution was plated onto Reasoner's 2A agar, TSA, and LB agar and incubated in darkness at room temperature for 4 d before microbial growth was assessed. The substrate‐filled syringes were flushed with 40 ml of sterile H_2_O followed by 40 ml of ½MS. Next, surface‐sterilised tomato seeds (MoneyMaker) were placed into each syringe, and six syringes were placed into an autoclaved Microbox container (SacO_2_, Deinze, Belgium) and placed in the glasshouse at 22°C during 16 h : 8 h day/night periods with a maximum of 80% relative humidity. After 2 wk, tomato plants were carefully uprooted under sterile conditions and inoculated with 10^6^ conidiospores ml^−1^ of either wild‐type TO22 or the corresponding *Av2* deletion strain. Subsequently, the plants were placed back into the syringe and the syringes into the container in the glasshouse. Symptoms of disease were scored at 14 dpi. For biomass quantification, stems of the plants were frozen in liquid nitrogen and ground to a fine powder. DNA was isolated using phenol–chloroform extraction (Chavarro‐Carrero *et al*., 2021). *Verticillium dahliae* biomass was quantified through real‐time PCR using *V. dahliae*‐specific primers targeting the ITS region of the ribosomal DNA. The tomato *Rubisco* gene was used for sample calibration.

## Results

### Av2 selectively inhibits bacterial growth *in vitro*

Most functionally characterised effectors target host physiology and are strictly *in planta* expressed, while microbiota‐manipulating effectors can be expressed *in planta* as well as during fungal life cycle stages outside the plant host (Snelders *et al*., 2020, 2021). In order to functionally characterise *Av2*, its expression was analysed by querying previously generated RNA sequencing datasets (de Jonge *et al*., 2012; Cook *et al*., 2020), revealing that *Av2* is not only expressed during host colonisation (1695 transcripts per million (TPM), 16, de Jonge *et al*., 2012) but also during *in vitro* growth on PDA (3256 TPM, 4 d old, Cook *et al*., 2020). Furthermore, *Av2* is expressed in conditions mimicking soil colonisation (Supporting Information Fig. S1). A similarly broad expression pattern, including expression in soil, has previously been observed for the *V. dahliae Ave1* effector gene (Fig. S1, Snelders *et al*., 2020), suggesting that Av2 may act as an antimicrobial too. Interestingly, *in silico* analysis using the Antimicrobial Peptide Scanner (v.2; Veltri *et al*., 2018) predicted antimicrobial activity for Av2 with a probability of 99.6%.

**Fig. 1:**
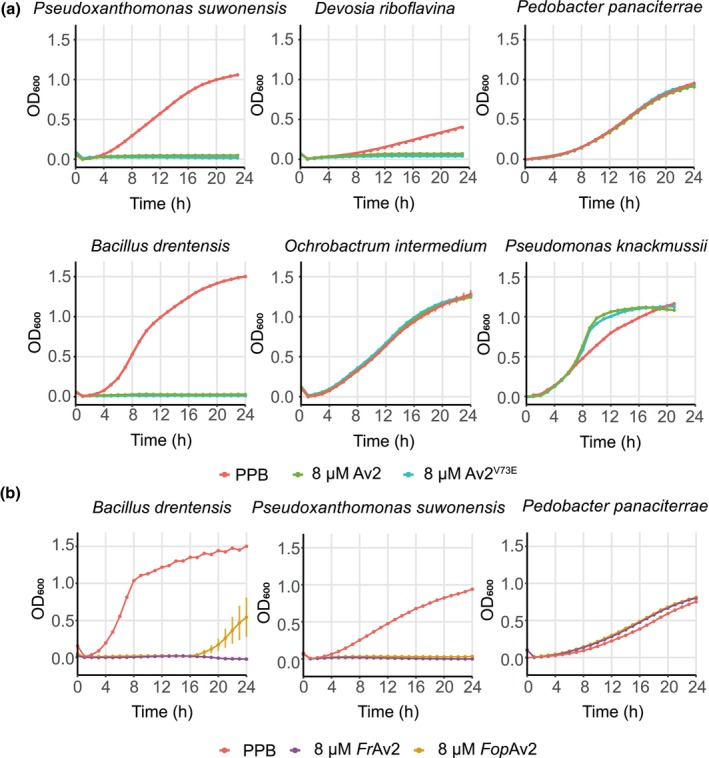
Av2 effector variants from *Verticillium dahliae* and homologues from *Fusarium* spp. display selective antibacterial activity. (a) The Av2 effector as well as the effector variant Av2^V73E^ selectively inhibits growth in a panel of phylogenetically diverse plant‐associated bacteria *in vitro*. (b) Av2 homologues from *Fusarium redolans* (*Fr*Av2) and *F. oxysporum* f. sp. *pisi* (*Fop*Av2) display an overlapping activity spectrum with the *V. dahliae* Av2 variants. Phosphate buffer (PPB) was used as a control. Graphs display time‐course measurements of bacterial densities in the presence or absence of effector proteins with 15 min intervals over 24 h and display the average OD_600_ of three biological replicates ± SD.

To validate the predicted antimicrobial activity of Av2 *in vitro*, the two previously identified variants, Av2 and Av2^V73E^, were expressed heterologously in *E. coli*, purified, and used in antimicrobial activity assays. Additionally, Av2 homologues from two *Fusarium* spp. were produced, purified, and tested for antimicrobial activity as well. To this end, a panel of 10 phylogenetically diverse plant‐associated bacteria was incubated with either of the Av2 variants at a concentration of 8 μM, or buffer as a control, and bacterial growth was assessed. Interestingly, three out of 10 bacteria showed reduced growth when incubated with either of the two *V. dahliae* Av2 variants, namely *Bacillus drentensis*, *Pseudoxanthomonas suwonensis*, and *Devosia riboflavina* (Fig. 1a). A subset of bacteria was also tested with the Av2 homologues from *Fusarium*, which showed activity against *Bacillus drentensis*, *Pseudoxanthomonas suwonensis* while *Devosia riboflavina* was not tested (Fig. 1b). Importantly, no differences in inhibitory activity were observed between any of the Av2 variants, including the homologues, suggesting they have overlapping activity spectra. Thus, all Av2 proteins display selective antimicrobial activity against bacteria *in vitro*.

To explore the potential mode of action of Av2, InterProScan was used to query for functional domains, but no such domains were detected. Furthermore, the structure of Av2 was predicted using AlphaFold2, resulting in a structural model with a low confidence score (pLDDT = 53.8), indicating limited reliability of the predicted structure (Fig. S2a). Nevertheless, FoldSeek was used to search for structural similarities between Av2 and previously characterised proteins, but no significant structural homologues were identified. These results suggest that, in addition to sharing sequence similarity only with *Fusarium* homologues, Av2 lacks detectable structural similarity to any known protein in the AlphaFold Protein Structure Database. To further investigate whether any compositional features could provide functional insight, the amino acid composition of Av2 was compared to that of other secreted proteins of *V. dahliae* (Fig. S2c). Consistent with the structural model that revealed positively charged surface regions (Fig. S2b), Av2 displayed a net positive charge of +2.33, in contrast to the average net charge of −10.1 for the secretome.

### *Av2* contributes to *V. dahliae* virulence through microbiota manipulation

**Fig. 2:**
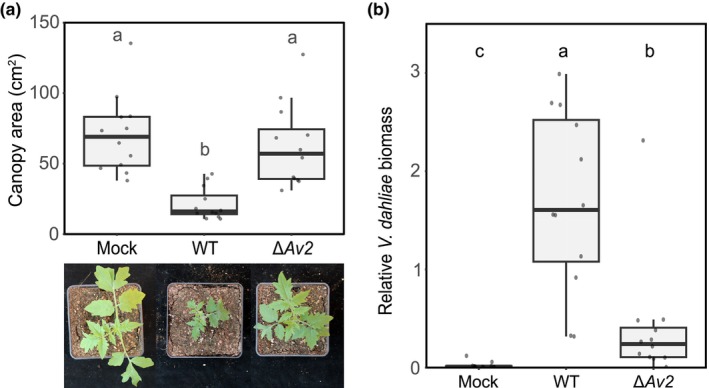
Av2 contributes to *Verticillium dahliae* virulence on tomato. (a) Canopy area measurements of inoculated plants show stronger stunting upon inoculation with wild‐type *V. dahliae* strain TO22 (WT) compared with the corresponding *Av2* deletion strain (Δ*Av2*). Mock‐inoculated plants were treated with sterile water. Different letters represent significant differences (one‐way ANOVA and Tukey's *post hoc* test; *P* < 0.05). Boxes indicate the interquartile range of the values, the median values are indicated by horizontal lines, and the whiskers extend to the minimum and maximum values. (b) *Verticillium dahliae* biomass in tomato stems was quantified with real‐time PCR and normalised to *Rubisco* abundance. Different letters represent significant differences (one‐way ANOVA and Tukey's *post hoc* test; *P* < 0.05).

Next, we hypothesised that *Av2* is utilised by *V. dahliae* for microbiota manipulation during host colonisation as well as during soil‐colonising stages. To investigate this hypothesis, we pursued microbiota sequencing through bacterial 16S ribosomal DNA profiling of tomato plants. To this end, tomato plants were inoculated with either wild‐type *V. dahliae* strain TO22 or the corresponding *Av2* deletion strain (Chavarro‐Carrero *et al*., 2021), while water treatment was used as a control. Interestingly, while tomato plants inoculated with wild‐type *V. dahliae* showed severely stunted growth by 10 d after inoculation compared with mock‐inoculated plants (Fig. 2a), plants inoculated with the *Av2* deletion strain only showed mild symptoms of disease, and significantly less stunting occurred than in plants inoculated with the wild‐type fungus. Importantly, significantly more fungal biomass was recorded in tomato plants inoculated with the wild‐type fungus than in plants inoculated with the *Av2* deletion strain (Fig. 2b), showing that *Av2* contributes to *V. dahliae* virulence during host colonisation.

**Fig. 3:**
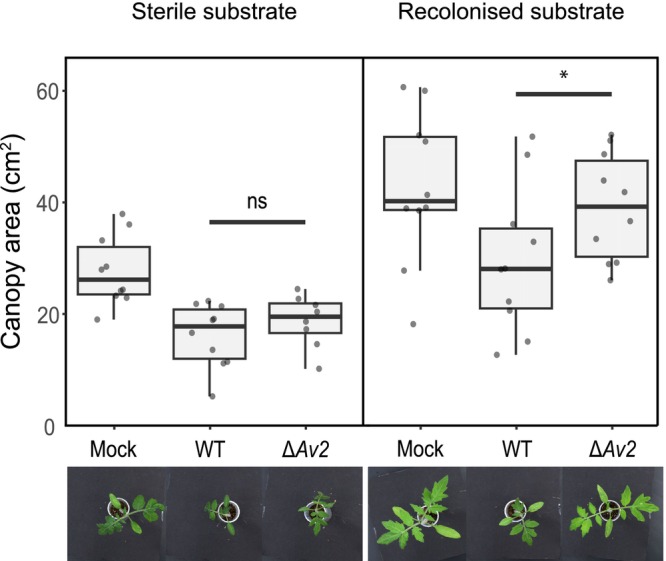
Av2 contributes to *Verticillium dahliae* virulence on tomato plants solely in the presence of microbes. Canopy area measurements of inoculated tomato plants grown in Flowpots show stronger stunting upon inoculation with wild‐type *V. dahliae* strain TO22 (WT) compared with the corresponding *Av2* deletion strain (Δ*Av2*) in recolonised substrate but not in sterile substrate. Mock‐inoculated plants were treated with sterile water. Statistical analyses were performed for each of the substrates, and the star indicates significant differences, whereas ns indicates no significant difference (unpaired two‐sided Student's *t*‐test; *P* < 0.05). Boxes indicate the interquartile range of the values, the median values are indicated by horizontal lines, and the whiskers extend to the minimum and maximum values. Photographs display phenotypes of representative plants for each of the treatments at 14 d post inoculation.

To address the hypothesis that *Av2* contributes to virulence through microbiota manipulation, tomato plants were inoculated in a peat‐based gnotobiotic system (Punt *et al*., 2025). If microbiota manipulation is the genuine function of the effector, *Av2* should not contribute to fungal virulence when plants are grown axenically, in the absence of microbes, while its contribution should become noticeable upon microbial reintroduction. To reintroduce microbes into sterile soil while maintaining physico‐chemical properties similar to the sterilised substrate, 10% unsterilised soil was mixed with 90% sterilised soil. Importantly, plating confirmed that sterilisation effectively removed the microbial population from the substrate, whereas reintroduction resulted in microbial colonisation of the originally sterilised substrate (Fig. S3). Next, tomato seedlings were inoculated with wild‐type *V. dahliae* strain TO22 or the corresponding *Av2* deletion strain and cultivated in the two substrates. Importantly, at 2 wk after inoculation, tomato plants inoculated with wild‐type *V. dahliae* were significantly smaller than the mock‐inoculated plants, while plants inoculated with the *Av2* deletion strain developed similar to tomato plants grown in potting soil (Figs 2a, 3), showing that *V. dahliae* can establish infections on tomato plants also in a gnotobiotic system on sterilised substrate. As previously observed for other plant species, tomato plants grown axenically generally developed slower than those grown in the presence of a microbiota on recolonised substrate (Kremer *et al*., 2021; Punt *et al*., 2025). However, when tomato plants were grown on sterile substrate, no difference could be observed between tomato plants inoculated with wild‐type *V. dahliae* or with the *Av2* deletion strain, showing that *Av2* only contributes to virulence in the presence of a microbiota. This finding suggests that microbiota manipulation is the genuine virulence function of the Av2 effector, and that the effector lacks plant virulence targets.

### *Av2* suppresses the recruitment of Pseudomonadales

**Fig. 4:**
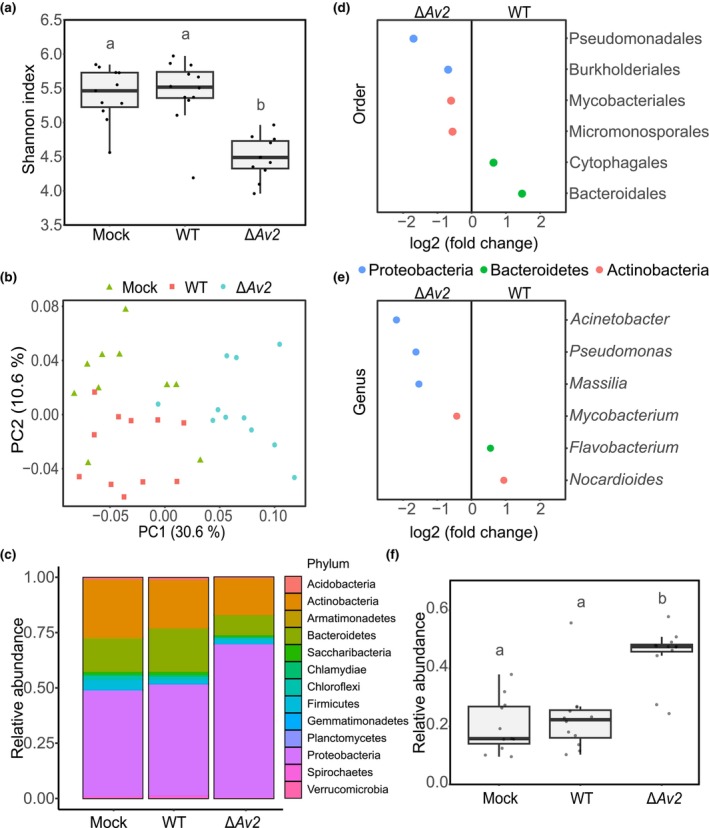
*Verticillium dahliae* Av2 suppresses *Pseudomonas* recruitment during host colonisation. (a) α‐diversity of tomato endosphere microbiota 10 d after inoculation as determined with 16S ribosomal DNA profiling. The α‐diversity is significantly lower for microbiomes of plants inoculated with the *Av2* deletion strain (Δ*Av2*) compared with the other treatments. Different letters represent significant differences (one‐way ANOVA and Tukey's *post hoc* test; *P* < 0.05). Boxes indicate the interquartile range of the values, the median values are indicated by horizontal lines, and the whiskers extend to the minimum and maximum values. (b) PCA based on weighted unifrac distance reveals separation of tomato stem endosphere microbiota upon inoculation with either water (Mock), wild‐type *V. dahliae*, or the *Av2* deletion strain (PERMANOVA, *P* < 0.001). (c) Relative abundance of bacterial phyla shows increased Proteobacteria abundance in plants inoculated with the *Av2* deletion strain. (d) Differentially abundant bacterial orders in the stem endosphere of tomato plants upon inoculation with either wild‐type *V. dahliae* or the *Av2* deletion strain (Wald test, adjusted *P* < 0.05). (e) Differential abundance analysis of bacteria at the genus level in the tomato stems upon inoculation with either wild‐type *V. dahliae* or the *Av2* deletion strain. (f) Relative abundance comparison of *Pseudomonas* in tomato stems upon inoculation with either water, wild‐type *V. dahliae*, or the *Av2* deletion strain. Different letters represent significant differences (one‐way ANOVA and Tukey's *post hoc* test; *P* < 0.05). The boxplot follows the same structures as in panel a.

To perform microbiota sequencing through bacterial 16S ribosomal DNA profiling, tomato stem samples were collected at 10 d post *V. dahliae* inoculation, before the onset of wilting symptoms, and the V5–V7 region of the bacterial 16S rDNA was amplified and sequenced. Subsequent analysis did not reveal major changes in microbial diversity (α‐diversity) between plants inoculated with *V. dahliae* wild‐type and mock‐inoculated plants (Fig. 4a). Interestingly, however, plants inoculated with the *V. dahliae Av2* deletion strain showed a significant reduction in microbial diversity that coincided with a strong increase in the relative abundance of Proteobacteria (Fig. 4c). PCA based on weighted unifrac distance revealed differential grouping of the tomato stem endosphere microbiota for the three different treatments (PERMANOVA, *P* < 0.001; Fig. 4b). To investigate which bacterial orders drove the separation of the samples in the PCA, pairwise bacterial abundance comparisons were performed between plants inoculated with *V. dahliae* wild‐type and the *Av2* deletion strain. Several bacterial orders were significantly more abundant in plants inoculated with the *Av2* deletion strain, namely Pseudomonadales, Burkholderiales, Mycobacteriales, and Micromonsporales, suggesting that these orders are particularly affected by the activity of the Av2 effector protein (Fig. 4d). Of these bacterial orders, the Pseudomonadales displayed the largest increase in abundance (log_2_‐fold change 1.67). Only a few genera appeared to drive the differential abundance of these bacterial orders. Within the Pseudomonadales, only the genera *Pseudomonas* and *Acinetobacter* were significantly more abundant upon inoculation with the *Av2* deletion strain, while within the order of Burkholderiales, only the genus *Massilia* showed a significant increase (Fig. 4e). The genus *Pseudomonas* especially caught our attention because of its high relative abundance in the tomato microbiota, with *c*. 20% and 50% in plants inoculated with the wild‐type *V. dahliae* and the *Av2* deletion strain, respectively. Intriguingly, while we anticipated a reduction in *Pseudomonas* abundance in plants inoculated with wild‐type *V. dahliae* compared with mock‐inoculated plants, we observed no difference in *Pseudomonas* abundance between the two treatments (Figs 4f, S5). This significant increase of *Pseudomonas* in plants inoculated with the *Av2* deletion strain also explains the decrease in alpha diversity of this treatment (Fig. 4a). Given that we only saw a strong recruitment of *Pseudomonas* during the infection by the *Av2* deletion strain, we conclude that this effector is utilised by *V. dahliae* to suppress the recruitment of this bacterial genus by the host upon pathogen invasion.

### *Verticillium dahliae* utilises Av2 to inhibit antagonistic *Pseudomonas* spp.

**Fig. 5:**
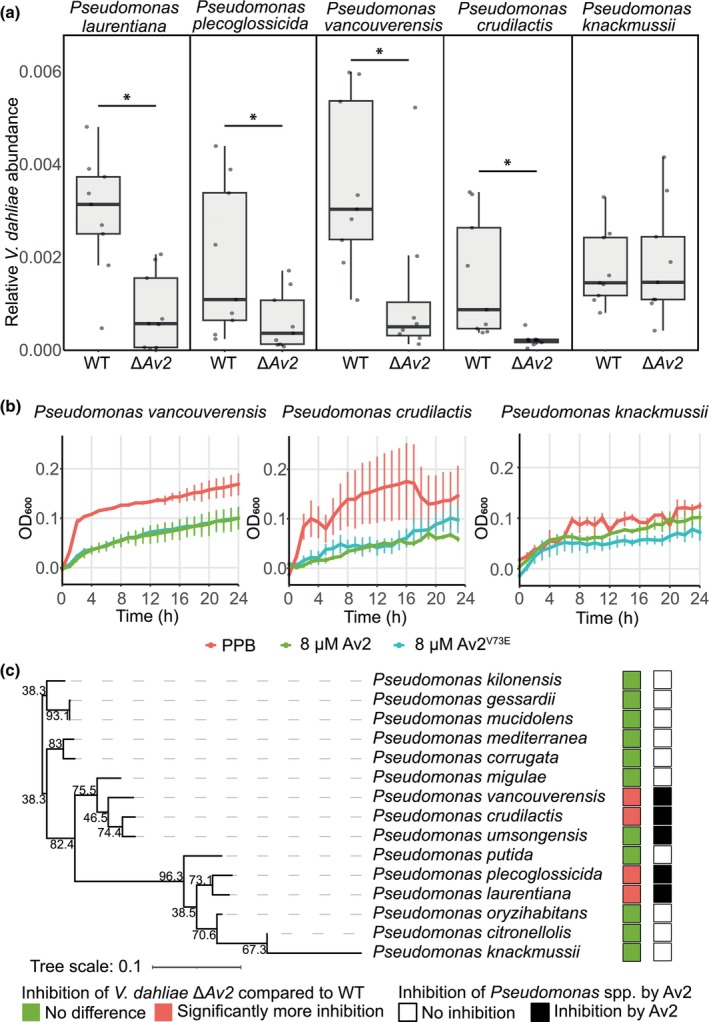
Av2 is used by *Verticillium dahliae* for direct growth inhibition of antagonistic *Pseudomonas* spp. (a) Relative biomass of wild‐type *V. dahliae* strain TO22 (WT) and the corresponding *VdAv2* deletion strain (Δ*Av2*) was quantified with real‐time PCR after cocultivation with a panel of Pseudomonadales in ½‐strength Murashige & Skoog medium for 48 h. The *V. dahliae* biomass was normalised against the abundance of spike‐in DNA, added during DNA extraction. Boxes indicate the interquartile range of the values, the median values are indicated by horizontal lines, and the whiskers extend to the minimum and maximum values. The asterisks indicate a significant difference in *V. dahliae* abundance between the genotypes (unpaired two‐sided Student's *t*‐test; *P* < 0.05). (b) *Pseudomonas* spp. are differentially inhibited by Av2 and Av2^V73E^ *in vitro*. Phosphate buffer (PPB) was used as the control. Graphs display time‐course measurements with 60 min intervals over 24 h and display the average OD_600_ of three biological replicates ± SD. (c) *Pseudomonas* spp. that display stronger antagonism towards the *Av2* deletion strain than towards wild‐type *V. dahliae* do not group in a phylogenetic tree that was generated based on 2495 orthologous genes present in all species. The numbers at the branches indicate bootstrap values. Inhibition of *Pseudomonas* spp. by Av2 and Av2^V73E^ *in vitro* is largely overlapping with that pattern.

The targeted recruitment of *Pseudomonas* by tomato plants upon *V. dahliae* colonisation, and the role of *Av2* in the prevention of such recruitment, suggests that *Pseudomonas* acts as an antagonist of the fungus. To investigate whether the interaction between *V. dahliae* and *Pseudomonas* involves direct antagonism and to elucidate the role of *Av2* in this interaction, competition assays were performed between *V. dahliae* and *Pseudomonas* strains isolated from tomato plants (Punt *et al*., 2025). To this end, wild‐type *V. dahliae* strain TO22 and the corresponding *Av2* deletion strain were incubated with a panel of 15 *Pseudomonas* species. Interestingly, wild‐type *V. dahliae* grew significantly better than the *Av2* deletion strain in the presence of any of the four *Pseudomonas* species *P. crudilactis*, *P. laurentiana*, *P. plecoglossicida*, or *P. vancouverensis* (Figs 5a, S4). No difference in growth between the two *V. dahliae* strains could be observed when cocultured with the remaining *Pseudomonas* species under these conditions. The reduced growth of the *Av2* deletion strain when cocultured with particular *Pseudomonas* species demonstrates that several *Pseudomonas* spp. are antagonists of *V. dahliae* growth and suggests that *Av2* is utilised by the fungus to counter these antagonists.

To test whether Av2 inhibits the growth of antagonistic *Pseudomonas* spp., their sensitivity towards Av2 was assessed *in vitro*. Interestingly, all antagonistic *Pseudomonas* spp. that showed reduced antagonism in the presence of *Av2* were inhibited when incubated with 8 μM Av2 or Av2^V73E^ (Fig. 5b). By contrast, most of the *Pseudomonas* spp. for which no difference in antagonism was recorded in the cocultivation with *V. dahliae* were unaffected by Av2 or Av2^V73E^, suggesting that *V. dahliae* coopted Av2 to selectively suppress antagonistic *Pseudomonas* spp. (Fig. 5c). To investigate the phylogenetic placement of the diverse *Pseudomonas* spp. isolates and assess potential clustering of the species that act as *V. dahliae* antagonists and are inhibited by Av2, 2495 orthologous genes present in all species were extracted from their genomic sequences and used to infer a phylogenetic tree. Interestingly, *Pseudomonas* spp. that showed increased antagonism towards the *Av2* deletion strain compared with wild‐type *V. dahliae* do not seem to cluster but appear in two clades (Fig. 5c). Further insight into the molecular function of Av2 could reveal whether this phylogenetic split is caused by the evolution of resistance against Av2 within the *Pseudomonas* genus or is due to physiological similarities among the inhibited antagonistic species. In conclusion, our findings suggest that *V. dahliae* exploits Av2 to suppress the cry for help recruitment of beneficial *Pseudomonas* spp. during plant colonisation.

## Discussion

The plant microbiota has been shown to be crucial for plant health and to act as an additional layer of defense against invading pathogens (Trivedi *et al*., 2020). In a phenomenon known as the ‘cry for help’, plants respond to pathogen invasion by dynamically altering their microbiota through modulating the composition of their root exudates to selectively recruit beneficial, disease‐suppressing microorganisms and thereby mitigate disease progression (Rolfe *et al*., 2019). Here, we characterise the *V. dahliae* effector *Av2* as an antimicrobial effector that actively suppresses the plant's cry for help. We show that in tomato, *Av2* suppresses the recruitment of antagonistic *Pseudomonas* spp. into the rhizosphere. As a result, plants inoculated with wild‐type *V. dahliae* exhibit *Pseudomonas* spp. levels comparable to mock‐inoculated controls, whereas infection with an *Av2*‐deletion mutant leads to strong *Pseudomonas* spp. enrichment that correlates with significantly reduced fungal colonisation. This activity is distinct from previously characterised antimicrobial effectors such as Ave1 and Ave1L2, which promote pathogen virulence by depleting antagonistic Sphingomonadales and Actinobacteria from the host plant microbiota (Snelders *et al*., 2020, 2023), or the suite of antimicrobial proteins secreted by *Albugo candida*, which collectively target core members of the *Arabidopsis thaliana* microbiota to facilitate host colonisation (Gómez‐Pérez *et al*., 2023). Although the overall structural model confidence is low, we observed positively charged surface areas which could point towards potential membrane activity, a mechanism previously described to various antimicrobial peptides (Oliveira Júnior *et al*., 2025). However, given the limited reliability of the structural prediction, this interpretation remains highly speculative. Our findings reveal a further sophisticated level of pathogen interference, demonstrating that pathogens can not only respond to and reshape the plant microbiome but also sabotage microbiota‐mediated host defence responses by compromising the cry for help recruitment during infection. *Pseudomonas* species are well known for their role in plant disease suppression and are frequently enriched during plant cry for help responses (Wang & Song, 2022). For example, beneficial *Pseudomonas* spp. are recruited in response to take‐all disease in wheat, where they protect the host through direct antagonism against the pathogen (Raaijmakers & Weller, 1998). We observed antagonism by *P. crudilactis*, *P. laurentiana*, *P. plecoglossicida* and *P. vancouverensis* against the *V. dahliae Av2* deletion mutant *in vitro*, indicating that these *Pseudomonas* spp. are capable of suppressing *V. dahliae* during infection. Furthermore, the same *Pseudomonas* spp. that exhibited enhanced antagonism towards the *Av2* deletion mutant were directly inhibited by Av2. This reciprocal antagonism aligns with previous findings showing that antimicrobial effectors target beneficial bacteria that are able to antagonise the pathogen (Snelders *et al*., 2020, 2023; Chavarro‐Carrero *et al*., 2024). Interestingly, *Pseudomonas* species inhibited by Av2 span two distinct phylogenetic groups, suggesting that some *Pseudomonas* species have evolved resistance to overcome suppression by Av2. This may suggest that a co‐evolutionary arms race takes place between *V. dahliae* and host‐associated microbiota members reminiscent of the development of antibiotic resistance. Given the abundance of microbes that produce antimicrobial molecules (Mullis *et al*., 2019; Mesny *et al*., 2025), the resistance of *Pseudomonas* spp. to Av2 may be part of a broader antimicrobial resistance developed through diverse microbial interactions, with *V. dahliae* playing only a minor role in this process. Elucidating how particular *Pseudomonas* species have overcome Av2 sensitivity may provide valuable insight into the mode of action of the effector and selective pressures shaping pathogen–microbe interactions in the rhizosphere. Within the *V. dahliae* population, two closely related homologues of the *Av2* effector have been identified, differing by only a single amino acid (Chavarro‐Carrero *et al*., 2021). Since this variation does not seem to affect recognition by the V2 immune receptor (Chavarro‐Carrero *et al*., 2021), we hypothesised that it might affect the antimicrobial activity that is exerted by the effector protein. However, our *in vitro* activity assays revealed no significant differences in antimicrobial activity between the two variants, suggesting that the amino acid substitution does not affect this function. In these assays, we also observed that the antimicrobial activity of Av2 extends beyond *Pseudomonas* spp., although it remains to be determined what the biological relevance of this broader activity is for *V. dahliae*. *Av2* homologues have furthermore been reported in other species of the *Verticillium* genus and in *Fusarium* (Chavarro‐Carrero *et al*., 2021). Intriguingly, recent evidence indicates that *V. dahliae* acquired *Av2* via horizontal gene transfer from *Fusarium* species (Sato *et al*., 2025). Although sequence variation exists among these homologues, our assays did not reveal any functional differences in antimicrobial activity. It is important to note, however, that only a limited panel of bacterial strains was tested, and the possibility remains that sequence variation modulates activity against untested microbial targets. The conservation of the antimicrobial function observed for Av2 is reminiscent of Ave1, which was also horizontally acquired by *V. dahliae*, in this case from plants (de Jonge *et al*., 2012; Snelders *et al*., 2020). Interestingly, plant homologues of *Ave1*, known as plant natriuretic peptides (PNP), likely exhibit similar antimicrobial activity *in vitro*, as both *A. thaliana* PNP‐A and Ave1 inhibit the growth of *Bacillus subtilis* (Snelders *et al*., 2020). These parallels raise the possibility that conserved antimicrobial effectors, regardless of their evolutionary origin, fulfil similar ecological roles in shaping microbial communities. Since both *Fusarium* spp. and *V. dahliae* are soil‐borne fungal pathogens that infect plants via the roots and disperse within their hosts via the vasculature (Di Pietro *et al*., 2003; Fradin & Thomma, 2006), further investigation into the role of *Av2* in *Fusarium* spp. could help clarify whether its conserved antimicrobial activity similarly contributes to the colonisation strategy shared by these pathogens.

Taken together, our findings broaden the understanding of how pathogens manipulate their hosts by revealing that antimicrobial effectors can actively suppress the pathogen‐induced cry for help response. By blocking the recruitment of protective microbes, pathogens undermine a critical layer of microbiota‐mediated immunity. This adds to growing evidence that the plant microbiota is a strategic battleground in host–pathogen interactions (Mesny *et al*., 2024). As more antimicrobial effectors are identified and characterised (Kettles *et al*., 2018; Snelders *et al*., 2020, 2021, 2023; Chang *et al*., 2021; Gómez‐Pérez *et al*., 2023; Ökmen *et al*., 2023; Chavarro‐Carrero *et al*., 2024; Mesny *et al*., 2025), it will become increasingly clear how deeply the molecular arms race between plants and pathogens extends into the plant's microbial sphere. Finally, given that the cry for help recruitment of beneficial microbes may ultimately lead to the establishment of disease‐suppressive soils (Mesny *et al*., 2024), future research will have to reveal whether Av2 suppresses such long‐term legacy effects in the soil microbiome.

## Competing interests

None declared.

## Author contributions

AK, WP, NCS, and BPHJT conceived the project. AK, WP, JZ, NCS, and BPHJT designed the experiments. AK, WP, AD, JZ, and NS performed the experiments. AK, WP, AD, JZ, NS, and BPHJT analysed the data. AK, WP, and BPHJT wrote the manuscript. All authors read and approved the final manuscript. AK and WP, and NCS and BPHJT contributed equally to this work.

## Disclaimer

The New Phytologist Foundation remains neutral with regard to jurisdictional claims in maps and in any institutional affiliations.

## Supporting information


**Fig. S1** *Verticillium dahliae Av2* is expressed in soil extract.
**Fig. S2** The predicted structure of the antimicrobial effector Av2 shows positively charged surface residues.
**Fig. S3** Microbes were successfully reintroduced into sterile flowpot substrate with 10% nonautoclaved soil.
**Fig. S4** Growth of a *Verticillium dahliae Av2* deletion strain is selectively impaired when cocultured with *Pseudomonas* spp.
**Fig. S5** Differentially abundant bacterial orders between mock and *Verticillium dahliae*‐inoculated plants.
Please note: Wiley is not responsible for the content or functionality of any Supporting Information supplied by the authors. Any queries (other than missing material) should be directed to the *New Phytologist* Central Office.

## Data Availability

The 16S profiling data have been deposited in the NCBI GenBank database under BioProject PRJEB90267.

## References

[nph70686-bib-0001] Berendsen RL , Vismans G , Yu K , Song Y , de Jonge R , Burgman WP , Burmølle M , Herschend J , H M Bakker PA , J Pieterse CM . 2018. Disease‐induced assemblage of a plant‐beneficial bacterial consortium. The ISME Journal 12: 1496–1507. 29520025 10.1038/s41396-018-0093-1 PMC5956071

[nph70686-bib-0002] Callahan BJ , McMurdie PJ , Rosen MJ , Han AW , Johnson AJA , Holmes SP . 2016. DADA2: high‐resolution sample inference from Illumina amplicon data. Nature Methods 13: 581–583. 27214047 10.1038/nmeth.3869 PMC4927377

[nph70686-bib-0003] Chang HX , Noel ZA , Chilvers MI . 2021. A β‐lactamase gene of *Fusarium oxysporum* alters the rhizosphere microbiota of soybean. The Plant Journal 106: 1588–1604. 33788336 10.1111/tpj.15257

[nph70686-bib-0004] Chavarro‐Carrero EA , Snelders NC , Torres DE , Kraege A , López‐Moral A , Petti GC , Punt W , Wieneke J , García‐Velasco R , López‐Herrera CJ *et al*. 2024. The soil‐borne white root rot pathogen *Rosellinia necatrix* expresses antimicrobial proteins during host colonization. PLoS Pathogens 20: e1011866. 38236788 10.1371/journal.ppat.1011866 PMC10796067

[nph70686-bib-0005] Chavarro‐Carrero EA , Vermeulen JP , Torres D , Usami T , Schouten HJ , Bai Y , Seidl MF , Thomma BPHJ . 2021. Comparative genomics reveals the *in planta*‐ secreted *Verticillium dahliae* Av2 effector protein recognized in tomato plants that carry the V2 resistance locus. Environmental Microbiology 23: 1941–1958. 33078534 10.1111/1462-2920.15288 PMC8246953

[nph70686-bib-0006] Cole JR , Wang Q , Fish JA , Chai B , McGarrell DM , Sun Y , Brown CT , Porras‐Alfaro A , Kuske CR , Tiedje JM . 2014. Ribosomal Database Project: data and tools for high throughput rRNA analysis. Nucleic Acids Research 42(D1): D633–D642. 24288368 10.1093/nar/gkt1244 PMC3965039

[nph70686-bib-0007] Cook DE , Kramer HM , Torres DE , Seidl MF , Thomma BPHJ . 2020. A unique chromatin profile defines adaptive genomic regions in a fungal plant pathogen. eLife 9: e62208. 10.7554/eLife.62208 PMC7781603 33337321

[nph70686-bib-0008] Cook DE , Mesarich CH , Thomma BPHJ . 2015. Understanding plant immunity as a surveillance system to detect invasion. Annual Review of Phytopathology 53: 541–563. 10.1146/annurev-phyto-080614-120114 26047564

[nph70686-bib-0009] Di Pietro A , Madrid MP , Caracuel Z , Delgado‐Jarana J , Roncero MIG . 2003. *Fusarium oxysporum*: exploring the molecular arsenal of a vascular wilt fungus. Molecular Plant Pathology 4: 315–325. 20569392 10.1046/j.1364-3703.2003.00180.x

[nph70686-bib-0010] Emms DM , Kelly S . 2019. OrthoFinder: phylogenetic orthology inference for comparative genomics. Genome Biology 20: 238. 31727128 10.1186/s13059-019-1832-y PMC6857279

[nph70686-bib-0011] Fadrosh DW , Ma B , Gajer P , Sengamalay N , Ott S , Brotman RM , Ravel J . 2014. An improved dual‐indexing approach for multiplexed 16S rRNA gene sequencing on the Illumina MiSeq platform. Microbiome 2: 1–7. 24558975 10.1186/2049-2618-2-6 PMC3940169

[nph70686-bib-0012] Faino L , Seidl MF , Shi‐Kunne X , Pauper M , Van Den Berg GCM , Wittenberg AHJ , Thomma BPHJ . 2016. Transposons passively and actively contribute to evolution of the two‐speed genome of a fungal pathogen. Genome Research 26: 1091–1100. 27325116 10.1101/gr.204974.116 PMC4971763

[nph70686-bib-0013] Fradin EF , Thomma BPHJ . 2006. Physiology and molecular aspects of Verticillium wilt diseases caused by *V. dahliae* and *V. albo‐atrum* . Molecular Plant Pathology 7: 71–86. 20507429 10.1111/j.1364-3703.2006.00323.x

[nph70686-bib-0014] Fradin EF , Zhang Z , Ayala JCJ , Castroverde CDM , Nazar RN , Robb J , Liu CM , Thomma BPHJ . 2009. Genetic dissection of Verticillium wilt resistance mediated by tomato Ve1. Plant Physiology 150: 320–332. 19321708 10.1104/pp.109.136762 PMC2675724

[nph70686-bib-0015] Gómez‐Pérez D , Schmid M , Chaudhry V , Hu Y , Velic A , Maček B , Ruhe J , Kemen A , Kemen E . 2023. Proteins released into the plant apoplast by the obligate parasitic protist Albugo selectively repress phyllosphere‐associated bacteria. New Phytologist 239: 2320–2334. 37222268 10.1111/nph.18995

[nph70686-bib-0016] Guo X , Zhang X , Qin Y , Liu YX , Zhang J , Zhang N , Wu K , Qu B , He Z , Wang X *et al*. 2020. Host‐associated quantitative abundance profiling reveals the microbial load variation of root microbiome. Plant Communications 1: 100003. 33404537 10.1016/j.xplc.2019.100003 PMC7747982

[nph70686-bib-0017] Jones JDG , Dangl JL . 2006. The plant immune system. Nature 444(Issue 7117): 323–329. 17108957 10.1038/nature05286

[nph70686-bib-0018] de Jonge R , Bolton MD , Kombrink A , Van Den Berg GCM , Yadeta KA , Thomma BPHJ . 2013. Extensive chromosomal reshuffling drives evolution of virulence in an asexual pathogen. Genome Research 23: 1271–1282. 23685541 10.1101/gr.152660.112 PMC3730101

[nph70686-bib-0019] de Jonge R , Van Esse HP , Maruthachalam K , Bolton MD , Santhanam P , Saber MK , Zhang Z , Usami T , Lievens B , Subbarao KV *et al*. 2012. Tomato immune receptor Ve1 recognizes effector of multiple fungal pathogens uncovered by genome and RNA sequencing. Proceedings of the National Academy of Sciences, USA 109: 5110–5115. 10.1073/pnas.1119623109 PMC3323992 22416119

[nph70686-bib-0020] Jumper J , Evans R , Pritzel A , Green T , Figurnov M , Ronneberger O , Tunyasuvunakool K , Bates R , Žídek A , Potapenko A *et al*. 2021. Highly accurate protein structure prediction with AlphaFold. Nature 596: 583–589. 34265844 10.1038/s41586-021-03819-2 PMC8371605

[nph70686-bib-0021] Jurrus E , Engel D , Star K , Monson K , Brandi J , Felberg LE , Brookes DH , Wilson L , Chen J , Liles K *et al*. 2018. Improvements to the APBS biomolecular solvation software suite. Protein Science 27: 112–128. 28836357 10.1002/pro.3280 PMC5734301

[nph70686-bib-0022] van Kempen M , Kim SS , Tumescheit C , Mirdita M , Lee J , Gilchrist CLM , Söding J , Steinegger M . 2023. Fast and accurate protein structure search with Foldseek. Nature Biotechnology 42: 243–246. 10.1038/s41587-023-01773-0 PMC10869269 37156916

[nph70686-bib-0023] Kettles GJ , Bayon C , Sparks CA , Canning G , Kanyuka K , Rudd JJ . 2018. Characterization of an antimicrobial and phytotoxic ribonuclease secreted by the fungal wheat pathogen *Zymoseptoria tritici* . New Phytologist 217: 320–331. 28895153 10.1111/nph.14786 PMC5724701

[nph70686-bib-0024] Kremer JM , Sohrabi R , Paasch BC , Rhodes D , Thireault C , Schulze‐Lefert P , Tiedje JM , He SY . 2021. Peat‐based gnotobiotic plant growth systems for Arabidopsis microbiome research. Nature Protocols 16: 2450–2470. 33911260 10.1038/s41596-021-00504-6 PMC10354810

[nph70686-bib-0025] Liu Y , Chen L , Wu G , Feng H , Zhang G , Shen Q , Zhang R . 2017. Identification of root‐secreted compounds involved in the communication between cucumber, the beneficial *Bacillus amyloliquefaciens*, and the soil‐borne pathogen *Fusarium oxysporum* . Molecular Plant–Microbe Interactions 30: 53–62. 27937752 10.1094/MPMI-07-16-0131-R

[nph70686-bib-0026] Liu Y , Zhang H , Wang J , Gao W , Sun X , Xiong Q , Shu X , Miao Y , Shen Q , Xun W *et al*. 2024. Nonpathogenic *Pseudomonas syringae* derivatives and its metabolites trigger the plant “cry for help” response to assemble disease suppressing and growth promoting rhizomicrobiome. Nature Communications 15: 1–14. 10.1038/s41467-024-46254-3 PMC10907681 38429257

[nph70686-bib-0027] López M , Tejera NA , Iribarne C , Lluch C , Herrera‐Cervera JA . 2008. Trehalose and trehalase in root nodules of *Medicago truncatula* and *Phaseolus vulgaris* in response to salt stress. Physiologia Plantarum 134: 575–582. 18823327 10.1111/j.1399-3054.2008.01162.x

[nph70686-bib-0028] Love MI , Huber W , Anders S . 2014. Moderated estimation of fold change and dispersion for RNA‐seq data with DESeq2. Genome Biology 15: 1–21. 10.1186/s13059-014-0550-8 PMC4302049 25516281

[nph70686-bib-0029] Martin M . 2011. Cutadapt removes adapter sequences from high‐throughput sequencing reads. EMBnet.Journal 17: 10–12.

[nph70686-bib-0030] McMurdie PJ , Holmes S . 2013. phyloseq: an R package for reproducible interactive analysis and graphics of microbiome census data. PLoS ONE 8: e61217. 23630581 10.1371/journal.pone.0061217 PMC3632530

[nph70686-bib-0031] Mesny F , Bauer M , Zhu J , Thomma BPHJ . 2024. Meddling with the microbiota: fungal tricks to infect plant hosts. Current Opinion in Plant Biology 82: 102622. 39241281 10.1016/j.pbi.2024.102622

[nph70686-bib-0032] Mesny F , Wolf V , López‐Moral A , Kraege A , Punt W , Park J , Zhu J , Sato Y , Thomma BP . 2025. Plant‐associated fungi co‐opt ancient antimicrobials for host manipulation. *BioRxiv* . doi: 10.1101/2024.01.04.574150. PMC13127573 42054466

[nph70686-bib-0033] Mullis MM , Rambo IM , Baker BJ , Reese BK . 2019. Diversity, ecology, and prevalence of antimicrobials in nature. Frontiers in Microbiology 10: 2518. 31803148 10.3389/fmicb.2019.02518 PMC6869823

[nph70686-bib-0034] Ökmen B , Katzy P , Huang L , Wemhöner R , Doehlemann G . 2023. A conserved extracellular Ribo1 with broad‐spectrum cytotoxic activity enables smut fungi to compete with host‐associated bacteria. New Phytologist 240: 1976–1989. 37680042 10.1111/nph.19244

[nph70686-bib-0035] Oksanen J , Simpson G , Blanchet F , Kindt R , Legendre P , Minchin P , O'Hara R , Solymos P , Stevens M , Szoecs E *et al*. 2004. vegan: community ecology package. [WWW document] URL https://github.com/vegandevs/vegan.

[nph70686-bib-0036] Oliveira Júnior NG , Souza CM , Buccini DF , Cardoso MH , Franco OL . 2025. Antimicrobial peptides: structure, functions and translational applications. Nature Reviews Microbiology 23: 687‐700. 10.1038/s41579-025-01200-y 40646173

[nph70686-bib-0037] Paulson JN , Colin Stine O , Bravo HC , Pop M . 2013. Differential abundance analysis for microbial marker‐gene surveys. Nature Methods 10: 1200–1202. 24076764 10.1038/nmeth.2658 PMC4010126

[nph70686-bib-0038] Punt W , Park J , Roevenich H , Kraege A , Schmitz N , Wieneke J , Snelders NC , Fiorin GL , López‐Moral A , Chavarro‐Carrero EA *et al*. 2025. A gnotobiotic system reveals multifunctional effector roles in plant‐fungal pathogen dynamics. *BioRxiv* . doi: 10.1101/2025.03.27.645772.

[nph70686-bib-0039] Raaijmakers JM , Weller DM . 1998. Natural plant protection by 2,4‐diacetylphloroglucinol‐producing Pseudomonas spp. in take‐all decline soils. Molecular Plant–Microbe Interactions 11: 144–152.

[nph70686-bib-0041] Raffaele S , Kamoun S . 2012. Genome evolution in filamentous plant pathogens: why bigger can be better. Nature Reviews Microbiology 10: 417–430. 22565130 10.1038/nrmicro2790

[nph70686-bib-0042] Rolfe SA , Griffiths J , Ton J . 2019. Crying out for help with root exudates: adaptive mechanisms by which stressed plants assemble health‐promoting soil microbiomes. Current Opinion in Microbiology 49: 73–82. 31731229 10.1016/j.mib.2019.10.003

[nph70686-bib-0043] Rovenich H , Boshoven JC , Thomma BPHJ . 2014. Filamentous pathogen effector functions: of pathogens, hosts and microbiomes. Current Opinion in Plant Biology 20: 96–103. 24879450 10.1016/j.pbi.2014.05.001

[nph70686-bib-0044] Sánchez‐Vallet A , Saleem‐Batcha R , Kombrink A , Hansen G , Valkenburg DJ , Thomma BPHJ , Mesters JR . 2013. Fungal effector Ecp6 outcompetes host immune receptor for chitin binding through intrachain LysM dimerization. eLife 2013: e00790. 10.7554/eLife.00790 PMC3700227 23840930

[nph70686-bib-0045] Sato Y , Bex R , van den Berg GCM , Santhanam P, Höfte M , Seidl MF , Thomma BPHJ . 2025. Starship giant transposons dominate plastic genomic regions in a fungal plant pathogen and drive virulence evolution. Nature Communications 16: 6806. 10.1038/s41467-025-61986-6 PMC12289983 40707455

[nph70686-bib-0046] Snelders NC , Boshoven JC , Song Y , Schmitz N , Fiorin GL , Rovenich H , van den Berg GCM , Torres DE , Petti GC , Prockl Z *et al*. 2023. A highly polymorphic effector protein promotes fungal virulence through suppression of plant‐associated Actinobacteria. New Phytologist 237: 944–958. 36300791 10.1111/nph.18576

[nph70686-bib-0047] Snelders NC , Petti GC , van den Berg GCM , Seidl MF , Thomma BPHJ . 2021. An ancient antimicrobial protein co‐opted by a fungal plant pathogen for *in planta* mycobiome manipulation. Proceedings of the National Academy of Sciences, USA 118: e2110968118. 10.1073/pnas.2110968118 PMC8670511 34853168

[nph70686-bib-0048] Snelders NC , Rovenich H , Petti GC , Rocafort M , van den Berg GCM , Vorholt JA , Mesters JR , Seidl MF , Nijland R , Thomma BPHJ . 2020. Microbiome manipulation by a soil‐borne fungal plant pathogen using effector proteins. Nature Plants 6: 1365–1374. 33139860 10.1038/s41477-020-00799-5

[nph70686-bib-0049] Spooren J , van Bentum S , Thomashow LS , Pieterse CMJ , Weller DM , Berendsen RL . 2024. Plant‐driven assembly of disease‐suppressive soil microbiomes. Annual Review of Phytopathology 62: 1–30. 10.1146/annurev-phyto-021622-100127 38857541

[nph70686-bib-0050] Torres DE , Kramer HM , Tracanna V , Fiorin GL , Cook DE , Seidl MF , Thomma BPHJ . 2024. Implications of the three‐dimensional chromatin organization for genome evolution in a fungal plant pathogen. Nature Communications 15: 1701. 10.1038/s41467-024-45884-x PMC10894299 38402218

[nph70686-bib-0051] Torres DE , Thomma BPHJ , Seidl MF . 2021. Transposable elements contribute to genome dynamics and gene expression variation in the fungal plant pathogen *Verticillium dahliae* . Genome Biology and Evolution 13: evab135. 34100895 10.1093/gbe/evab135 PMC8290119

[nph70686-bib-0052] Trivedi P , Leach JE , Tringe SG , Sa T , Singh BK . 2020. Plant–microbiome interactions: from community assembly to plant health. Nature Reviews Microbiology 18: 607–621. 32788714 10.1038/s41579-020-0412-1

[nph70686-bib-0053] Usami T , Momma N , Kikuchi S , Watanabe H , Hayashi A , Mizukawa M , Yoshino K , Ohmori Y . 2017. Race 2 of *Verticillium dahliae* infecting tomato in Japan can be split into two races with differential pathogenicity on resistant rootstocks. Plant Pathology 66: 230–238.

[nph70686-bib-0054] Varadi M , Anyango S , Deshpande M , Nair S , Natassia C , Yordanova G , Yuan D , Stroe O , Wood G , Laydon A *et al*. 2022. AlphaFold Protein Structure Database: massively expanding the structural coverage of protein‐sequence space with high‐accuracy models. Nucleic Acids Research 50(D1): D439–D444. 34791371 10.1093/nar/gkab1061 PMC8728224

[nph70686-bib-0055] Veltri D , Kamath U , Shehu A . 2018. Deep learning improves antimicrobial peptide recognition. Bioinformatics 34: 2740–2747. 29590297 10.1093/bioinformatics/bty179 PMC6084614

[nph70686-bib-0056] Wang Z , Song Y . 2022. Toward understanding the genetic bases underlying plant‐mediated “cry for help” to the microbiota. iMeta 1: e8. 38867725 10.1002/imt2.8 PMC10989820

[nph70686-bib-0057] Wippel K , Tao K , Niu Y , Zgadzaj R , Kiel N , Guan R , Dahms E , Zhang P , Jensen DB , Logemann E *et al*. 2021. Host preference and invasiveness of commensal bacteria in the Lotus and Arabidopsis root microbiota. Nature Microbiology 6: 1150–1162. 10.1038/s41564-021-00941-9 PMC8387241 34312531

[nph70686-bib-0058] Yuan J , Zhao J , Wen T , Zhao M , Li R , Goossens P , Huang Q , Bai Y , Vivanco JM , Kowalchuk GA *et al*. 2018. Root exudates drive the soil‐borne legacy of aboveground pathogen infection. Microbiome 6: 1–12. 30208962 10.1186/s40168-018-0537-x PMC6136170

