# Supplementary material for: Undermining the cry for help: the phytopathogenic fungus Verticillium dahliae secretes an antimicrobial effector protein to undermine host recruitment of antagonistic Pseudomonas bacteria
Source: New Phytol. 2025 Oct 29;249(1):406–17. doi: 10.1111/nph.70686 (PMC12676067; doi:10.1111/nph.70686)
Supplement: Supplementary file 1 — Fig. S1 Verticillium dahliae Av2 is expressed in soil extract. Fig. S2 The predicted structure of the antimicrobial effector Av2 shows positively charged surface residues. Fig. S3 Microbes were successfully reintroduced into sterile flowpot substrate with 10% nonautoclaved soil. Fig. S4 Growth of a Verticillium dahliae Av2 deletion strain is selectively impaired when cocultured with Pseudomonas spp. Fig. S5 Differentially abundant bacterial orders between mock and Verticillium dahliae‐inoculated plants. Please note: Wiley is not responsible for the content or functionality of any Supporting Information supplied by the authors. Any queries (other than missing material) should be directed to the New Phytologist Central Office. [file NPH-249-406-s001.pdf]

New Phytologist Supporting Information

Article title: **Undermining the cry for help: The phytopathogenic fungus *Verticillium dahliae* secretes an antimicrobial effector protein to undermine host recruitment of antagonistic *Pseudomonas* bacteria**

Authors: Anton Kraege[1,#], Wilko Punt[1,#], Andrea Doddi[1,2], Jinyi Zhu[1], Natalie Schmitz[1], Nick C. Snelders[1,3,$], Bart P.H.J. Thomma[1,$,*]

[1]University of Cologne, Institute for Plant Sciences, Cluster of Excellence on Plant Sciences (CEPLAS), 50674 Cologne, Germany;
[2]Sapienza University of Rome, Department of Environmental Biology, 00185 Rome, Italy;
[3]University of Utrecht, Theoretical Biology and Bioinformatics Group, Department of Biology, 3584CH Utrecht, The Netherlands;

[#]These authors contributed equally
[$]These authors contributed equally
[*]To whom correspondence should be addressed. E-mail: bthomma@uni-koeln.de

Article acceptance date: 8 October 2025

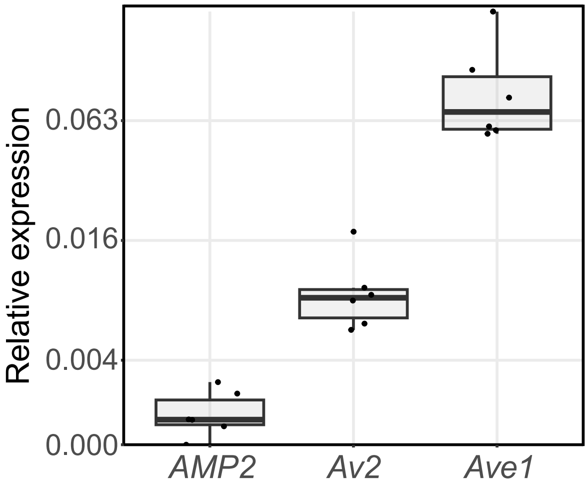

Supplementary Fig. 1. *Verticillium dahliae* Av2 is expressed in soil extract. Expression of *V. dahliae* effectors after seven days of growth in soil extract when normalised to glyceraldehyde 3-phosphate dehydrogenase (*VdGAPDH*) expression. Boxes indicate the interquartile range of the values, the median values are indicated by horizontal lines, and the whiskers extend to the minimum and maximum values.

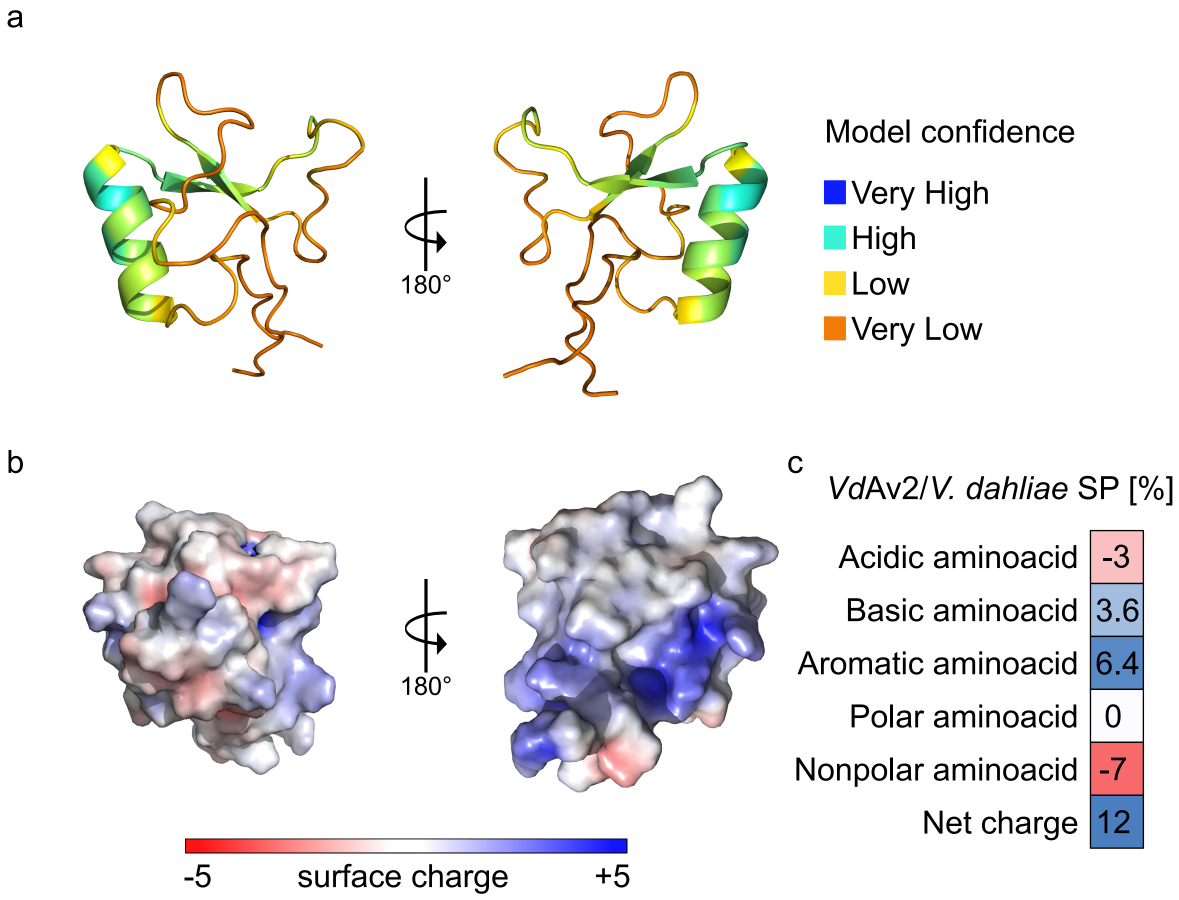

Supplementary Fig. 2. The predicted structure of the antimicrobial effector Av2 shows positively charged surface residues. (a) Structural prediction of Av2 using Alphafold2 resulted in a low confidence structure with an overall pLDDT score of 53.8. Colouring of the individual amino acids in the structure according to the Alphafold Protein Structure Database, where amino acids with a pLDDT score >90 appear in blue, between 90 and 70 in cyan, between 70 and 50 in yellow and <50 in orange. While local stretches have higher pLDDT scores, the majority of Av2 is predicted with a score <50. **(b)** Surface charge of the predicted structure was calculated using the APBS plugin for PyMOL. **(c)** To assess differences in amino acid composition between Av2 and the average *V. dahliae* secreted protein, the proportion of chemically distinct amino acids in Av2 was subtracted from their expected proportions in the overall secretome.

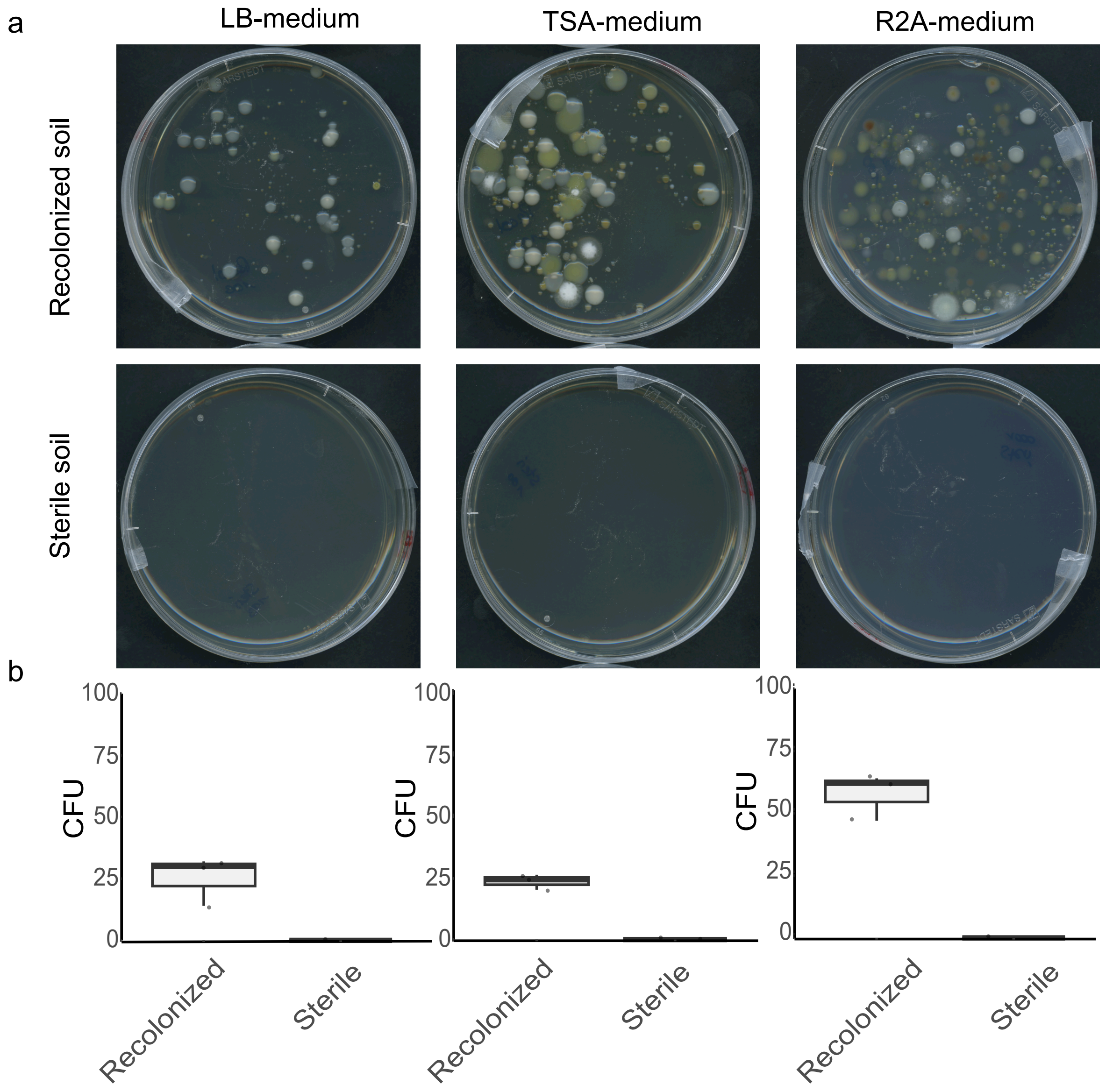

Supplementary Fig. 3. Microbes were successfully reintroduced into sterile flowpot substrate with 10% non-autoclaved soil. (a) Either recolonized or sterile Flowpot substrate were resuspended in MgCl2 and streaked out on three different media, namely Lysogeny broth agar (LB), Tryptic soy agar (TSA) and Reasoner's 2A agar (R2A). There was growth on all plates containing recolonised substrate while no growth was observed on plates with sterile substrate. Photographs display agar plates after the plating of a 100x diluted substrate-MgCl2 suspension and a 4-day incubation in darkness at room temperature. **(b)** Boxplots displaying the number of colony-forming units (CFU) on three different growth media after plating a 100x substrate-MgCl2 suspension and 4 days of incubation in darkness at room temperature. Substrate suspension from recolonized substrate showed significant more colonies compared to sterile substrate (unpaired two-sided student's t-test; $p < 0.05$). Boxes indicate the interquartile range of the values, the median values are indicated by horizontal lines, and the whiskers extend to the minimum and maximum values.

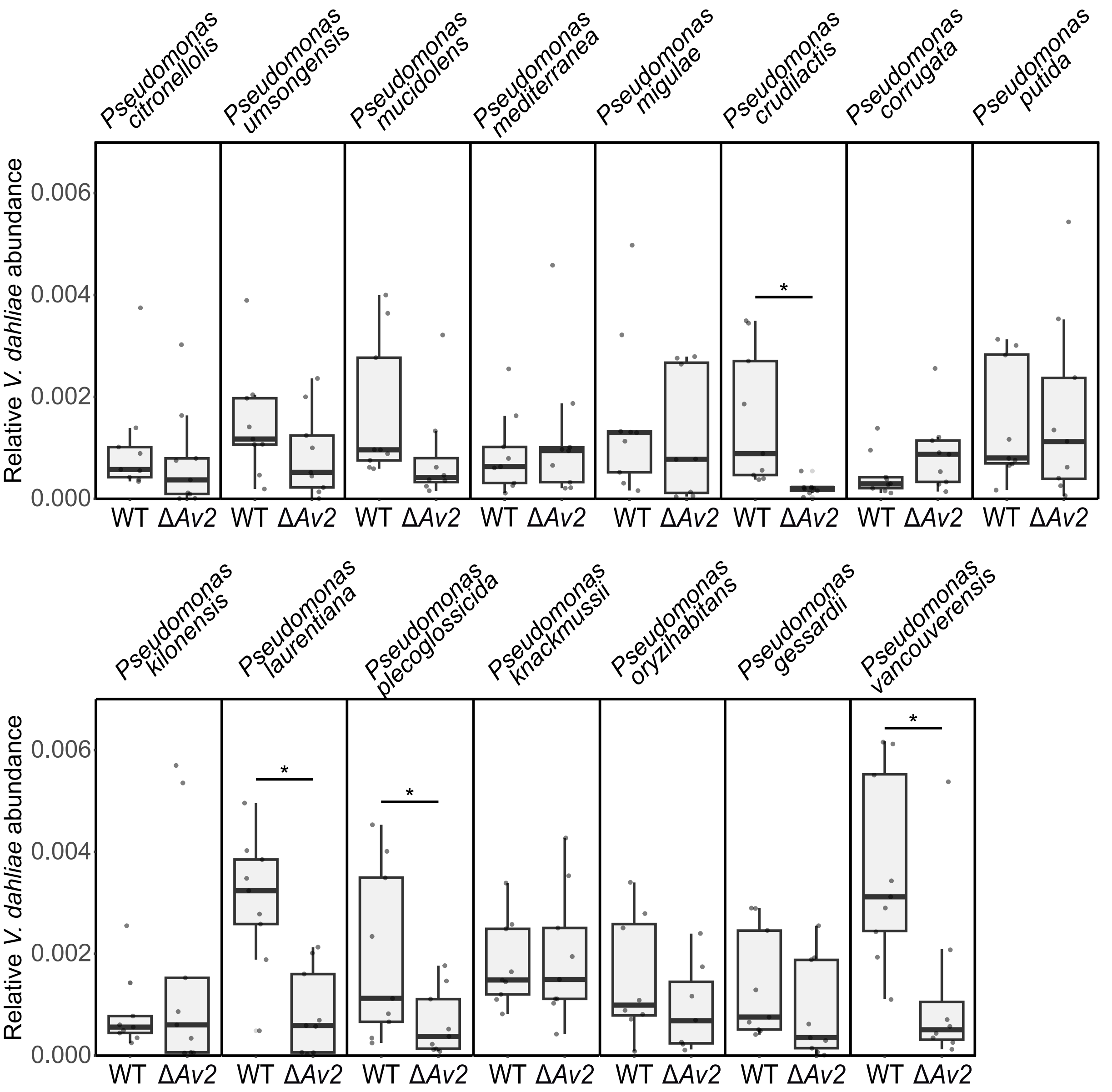

Supplementary Fig. 4. Growth of a *Verticillium dahliae Av2* deletion strain is selectively impaired when co-cultured with *Pseudomonas* spp. Relative biomass of wild-type *V. dahliae* strain TO22 (WT) and the corresponding *VdAv2* deletion strain (Δ*Av2*) was quantified with real-time PCR after co-cultivation with a panel of Pseudomonadales in half-strength Murashige and Skoog medium for 48 h. V. dahliae biomass was normalised against abundance of spike-in DNA added during DNA extraction. The asterisks indicate significant difference in V. dahliae abundance between the genotypes (unpaired two-sided student's t-test; $p < 0.05$). Boxes indicate the interquartile range of the values, the median values are indicated by horizontal lines, and the whiskers extend to the minimum and maximum values.

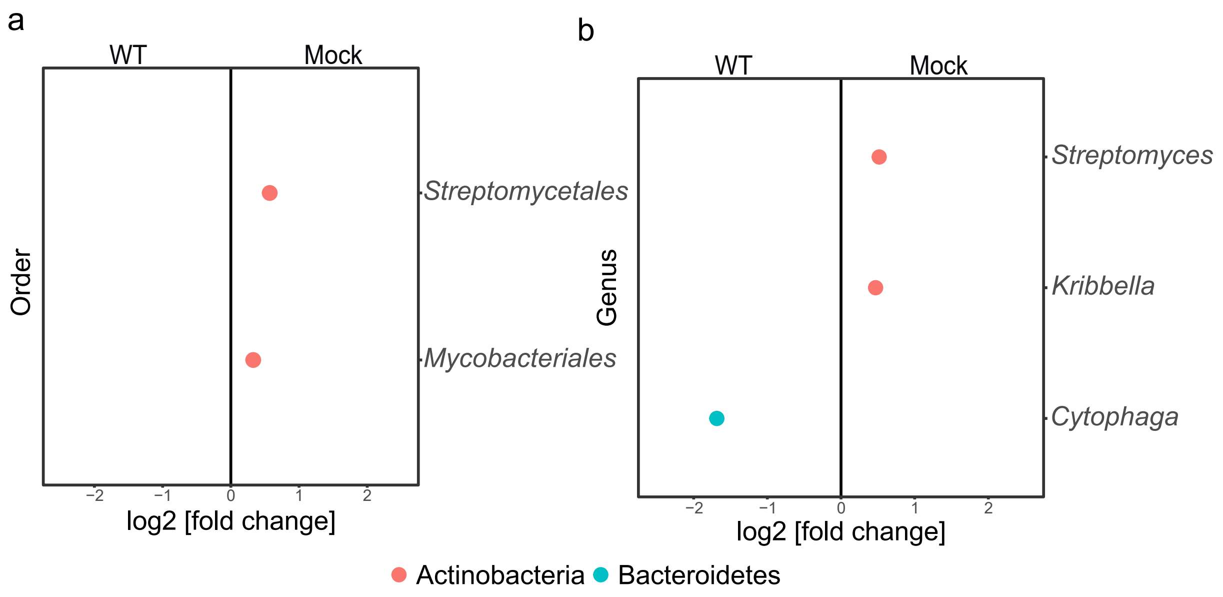

Supplementary Fig. 5. Differentially abundant bacterial orders between mock and *Verticillium dahliae* inoculated plants. (a) Differentially abundant bacterial orders in the stem endosphere of tomato plants upon inoculation with either wild-type V. dahliae (WT) or mock treatment (Wald test, adjusted $P < 0.05$). **(b)** Differential abundance analysis of bacterial genera in the tomato stems upon inoculation with wild-type *V. dahliae* or mock treatment.
